# Interleukin-6 Contributes to Age-Related Alteration of Cytokine Production by Macrophages

**DOI:** 10.1155/2010/475139

**Published:** 2010-07-07

**Authors:** Christian R. Gomez, John Karavitis, Jessica L. Palmer, Douglas E. Faunce, Luis Ramirez, Vanessa Nomellini, Elizabeth J. Kovacs

**Affiliations:** ^1^Department of Surgery, and Immunology and Aging Program, The Burn and Shock Trauma Institute, Loyola University Medical Center, 2160 South First Avenue, Maywood, IL 60153, USA; ^2^Department of Cell Biology, Neurobiology and Anatomy and Alcohol Research Program, Loyola University Medical Center, 2160 South First Avenue, Maywood, IL 60153, USA; ^3^Stritch School of Medicine, Loyola University Medical Center, 2160 South First Avenue, Maywood, IL 60153, USA

## Abstract

Here, we studied *in vitro* cytokine production by splenic macrophages obtained from young and aged BALB/c wild type (WT) and IL-6 knockout (IL-6 KO) mice. Relative to macrophages obtained from young WT mice given lipopolysaccharide (LPS), those from aged WT mice had decreased production of proinflammatory cytokines. In contrast, when compared to macrophages from young IL-6 KO mice, LPS stimulation yielded higher levels of these cytokines by cells from aged IL-6 KO mice. Aging or IL-6 deficiency did not affected the percentage of F4/80^+^ macrophages, or the surface expression of Toll-like receptor 4 (TLR4) and components of the IL-6 receptor. Overall, our results indicate that IL-6 plays a role in regulating the age-related defects in macrophages through alteration of proinflammatory cytokines, adding to the complexity of IL-6-mediated impairment of immune cell function with increasing age.

## 1. Introduction

As a part of the age-associated deterioration of the immune system [1–3], there is a chronic proinflammatory state even in the absence of clinically-apparent disease. This state defined as “inflamm-aging” is characterized by elevated circulating levels of proinflammatory factors (IL-1*β*, IL-6, TNF*α*, and prostaglandin E_2_) and anti-inflammatory mediators, (IL-1 receptor antagonist, soluble TNF receptor, IL-10, transforming growth factor beta, acute phase proteins, C-reactive protein, and serum amyloid A) and contributes to the decreased ability of the elderly to mount an appropriate immune response following an infectious challenge [4–7].

One of the most prominent aspects of “inflamm-aging” is in presence of elevated circulating levels of the proinflammatory cytokine, interleukin (IL)-6 [8, 9]. In an attempt to better define the role of IL-6 in the systemic inflammatory response in the aged, we gave young and aged wild type (WT) and IL-6 knockout (KO) BALB/c female mice lipopolysaccharide (LPS). Following the inflammatory challenge, aged IL-6 KO mice had improved survival when compared to aged WT mice [10]. Moreover, we previously reported that aged IL-6 KO mice showed a decreased acute phase response when compared to that of aged WT mice [10]. In addition, hepatic injury was drastically reduced in aged IL-6 KO mice given LPS as compared with LPS-exposed aged WT mice [11]. Recently, Starr et al. reported similar findings in male C57BL/6 IL-6KO mice using a higher dose of LPS (5 mg/kg) and a slightly older group of mice (26 months old) [12] than our study. Taken together, these observations have suggested a crucial role for IL-6 as a component of the aberrant systemic innate immune responses of the aged after an inflammatory challenge or injury and that this role is not sex or strain specific.

Macrophages play critical roles in phagocytosis of antigens, microorganisms, and cellular debris; killing of invading pathogens and tumors; and wound healing [13]. Many of the macrophage functions are carried out by secreted cytokines, which in turn, regulate multiple immune functions, especially inflammatory responses [14]. The effects of aging on cytokine production by macrophages have shown conflicting results with *in vitro* stimulation of purified macrophage populations with LPS and *in vivo* systemic administration of LPS. For example, our laboratory and others reported that macrophages from aged mice produce less TNF*α* and IL-6 after *in vitro* exposure to LPS than comparably stimulated cells from young mice [15–19]. These findings have been observed despite the presence of “inflamm-aging” and the elevated baseline inflammatory state seen in healthy aged individuals [20, 21].

Since macrophages are considered to be an important source of proinflammatory cytokines *in vivo*, many possible explanations for this inconsistency can be proposed [22]. Among the multiple possibilities, the effect of the local environment *in vitro *versus the effect of the aged microenvironment *in vivo* has been considered [22–24]. We reported that serum from aged Fisher 344 rats increased the levels of IL-6 by macrophages obtained from young animals and cultured *in vitro* without stimulants [25]. These observations suggest that the elevated production of inflammatory mediators in the aged results, in part, from the interaction of macrophages with a variety of factors in their environment *in situ*.

To analyze the role that IL-6 might play in regulating the age-related defects in macrophages through alteration of proinflammatory cytokines, we exposed splenic macrophages from young and aged WT and IL-6 KO mice to LPS. Our findings suggest that IL-6 regulates the age-related defects in macrophages through alteration of proinflammatory cytokines and that these alterations are not due to changes in surface expression of TLR4 or the IL-6 receptor.

## 2. Materials and Methods

### 2.1. Animals

Young (2 months old) and aged (18 months old) WT female BALB/c mice were purchased from the National Institute of Aging colony at Harlan Laboratories (Indianapolis, IN). IL-6 KO mice, kindly provided by Dr. Manfred Kopf, Molecular Biomedicine, ETH Zurich, Switzerland, have a disruption of IL-6 in the second exon (first coding exon) by insertion of a neo^r^ cassette [26]. IL-6 KO mice were backcrossed onto the BALB/c background, bred, maintained, and aged at the Taconic Laboratories (Germantown, NY). Young (2 months old) and aged (18 months old) female BALB/c IL-6 KO mice were used in the experiments described here. Animals were housed under similar conditions at their respective facilities. They were free of potential endemic viral pathogens that could influence their inflammatory response. All animals where maintained in an environmentally controlled facility at Loyola University Medical Center for at least one week prior to experimentation. At the time of sacrifice, all mice were dissected and the organs were screened for visible tumors and/or gross abnormalities. If found, these animals were removed from the study. The experimental protocols described here followed the guidelines established by the publication, Principles of Laboratory Animal Care (NIH publication no. 86–23, revised 1985), and were approved by the Loyola University Chicago Institutional Animal Care and Use Committee.

### 2.2. Cell Isolation and Culture

Mice were sacrificed by CO_2_ inhalation and subsequent cervical dislocation. In order to avoid confounding factors related to circadian rhythms, all animal protects were performed between 8 and 9 AM. Splenic macrophages were isolated by plastic adherence as previously described [17]. Briefly, spleens were aseptically removed and disrupted to yield a cell suspension in RPMI 1640 medium, supplemented with 5% FBS, penicillin (100 U/ml), streptomycin (100 *μ*g/ml), and 2 mM glutamine (culture medium) (GIBCO-BRL, Grand Island, NY). Following red blood cell lysis with ACK Lysis Buffer (Invitrogen Corp., Carlsbad, CA), white blood cells were counted in a hemocytometer and viability was determined by Trypan Blue exclusion. Two ×10^6^ cells were seeded in 96-well plates in 200 *μ*l culture medium. After incubation for 2 hours at 37°C and 5% CO_2_, nonadherent cells were discarded by medium aspiration and washed twice with warm phosphate buffer saline (PBS). This method resulted in an adherent population characterized as >98% positive for Mac-3 and Di-I-acetylated low-density lipoprotein uptake, as we previously reported [27]. Adherent cells were treated in 200 *μ*l culture medium alone or containing 100 ng/ml LPS from Escherichia coli 0111:B4 (Sigma Biosciences, St. Louis, MO). In the absence of stimulation, macrophage cytokine levels were undetectable ([16] and data not shown). Supernatants were collected after 16 hours and stored at −80°C.

### 2.3. Measurement of Proinflammatory Cytokines

The concentrations of TNF*α*, IL-1*β*, IL-6, and IL-12 in macrophage supernatants were measured by commercially available OptEIA ELISA kits (BD Pharmingen, San Diego,CA) according to the manufacturer instructions. The lowest detectable limit of these kits is 15.6 pg/ml. Data are expressed as pg/ml.

### 2.4. Flow Cytometry

Total spleen cell suspensions were obtained as reported above. Flow cytometry was performed as previously described [16, 17]. Briefly, after blocking nonspecific staining with anti-CD16/CD32 (Fc*γ*III/II; BD-PharMingen, San Diego, CA), total splenocyte suspensions were stained with APC-conjugated anti-CD3 (0.25 *μ*g/1 × 10^6^ cells, clone 145-2C11, eBioscience, San Diego, CA), PE-conjugated anti-CD4 (0.125 *μ*g/1 × 10^6^ cells, clone GK1.5, eBioscience, San Diego, CA), FITC-conjugated anti-CD8 (0.5 *μ*g/1 × 10^6^ cells, clone 53–6.7, eBioscience, San Diego, CA), FITC-conjugated anti-Ly49c (0.5 *μ*g/1 × 10^6^ cells, clone 5E6, eBioscience, San Diego, CA), Alexa fluor 750-conjugated anti-F4/80 (0.1 *μ*g/1 × 10^6^ cells, clone BM8, Invitrogen Corp., Camarillo, CA), PE-conjugated anti-IL-6Ra (0.06 *μ*g/1 × 10^6^ cells, clone D7715A7, BD-PharMingen, San Diego, CA), APC-conjugated anti-CD19 (0.5 *μ*g/1 × 10^6^ cells, clone MB 19–1, eBioscience, San Diego, CA), and biotin-conjugated anti-TLR4 (0.25 *μ*g/1 × 10^6^ cells, clone MTS510, eBioscience, San Diego, CA), biotin-conjugated anti-gp130 (1 *μ*g/1 × 10^6^ cells, clone 125623, R&D Systems Inc., Minneapolis, MN). Following biotin-conjugate incubation, cells were incubated with PerCP-conjugated streptavidin (0.125 *μ*g/1 × 10^6^ cells, eBioscience, San Diego, CA). Flow cytometric determinations were made using Becton Dickinson FACSCanto flow cytometer (BDIS, San Jose, CA) and data was analyzed with FlowJo software (Tree Star Inc., Ashland, OR).

### 2.5. Statistical Analysis

Data are expressed as mean ± SEM. ANOVA and Tukey-Kramer Multiple Comparisons were used to determine statistical significance using GraphPad Prism Version 2.0 statistical package (San Diego, CA, USA). A *P *value less than .05 was considered significant.

## 3. Results

### 3.1. Cytokine Levels in Macrophages from Aged IL-6-Deficient Mice after LPS Stimulation

**Figure 1:**
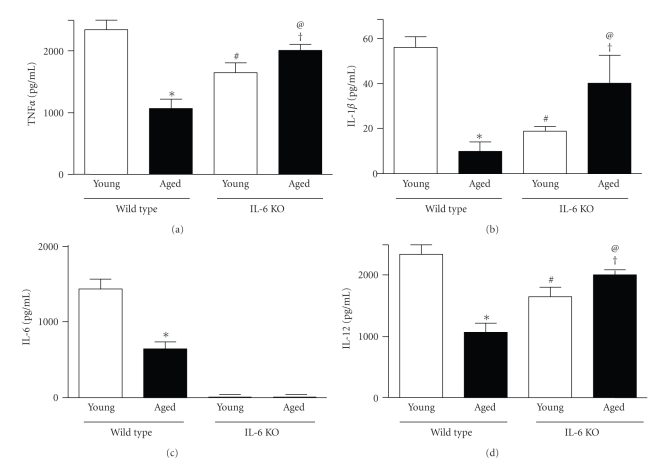
Splenic macrophages obtained from young and aged WT and IL-6 KO mice were cultured for 18 hours with LPS (100 ng/ml). Supernatants collected were assayed for TNF*α* (a), IL-1*β* (b), IL-6 (c), and IL-12 (d), by ELISA. Data are shown as mean ± SEM of 4-5 mice per group. *P < .05 from young WT. ^#^ *P* < .05 from young WT. ^†^ *P* < .05 from young IL-6 KO. ^@^ *P* < .05 from aged WT.

 When stimulated *in vitro* with LPS, macrophages from aged mice produce less cytokines than comparably stimulated cells from young mice [15–19]. To determine if this phenotype may be determined in part by IL-6, which is elevated in the circulation of healthy aged individuals [9, 10, 28, 29], we evaluated proinflammatory cytokine production in splenic macrophages obtained from young aged WT and IL-6 KO mice. Macrophages from aged and young mice produced proinflammatory cytokines upon LPS stimulation (Figures 1(a)–1(d)). As previously reported [15–19], macrophages from aged WT mice had decreased production of TNF*α* and IL-6 (55%), as well as IL-1*β* (80%) and IL-12 (35%) relative to splenic macrophages obtained from young WT mice given LPS. Similar to macrophages from aged WT mice, the production of cytokines by macrophages from young IL-6 KO mice was reduced for TNF*α* (67%), IL-1*β* (31%), and IL-12 (71%) relative to that of young WT mice (Figure 1). In contrast, as compared to macrophages from young IL-6 KO mice, LPS exposure induced higher cytokine production by cells from aged IL-6 KO mice (1.3, 2.2 and 1.2-fold for TNF*α*, IL-1*β* and IL-12, resp.) (Figure 1). Cytokine production by macrophages from aged IL-6 KO mice was elevated when compared to cells from aged WT mice (1.8, 3.7 and 1.9-fold for TNF*α*, IL-1*β* and IL-12, resp.). Overall, these results show that IL-6 plays a role in regulating the age-related defects in macrophages through alteration of the production of proinflammatory cytokines. 

### 3.2. Effects of Age and IL-6 on Splenocyte Cell Populations

Interleukin-6 is a pleiotropic cytokine, which has an important role in supporting the growth of T and B lymphocytes [30] in lymphoid tissues, mainly in the spleen [31]. Therefore, lack of expression of IL-6 could modify the phenotype of splenocytes and their production of cytokines when cultured *in vitro*. To test this hypothesis, total spleen cell suspensions were examined by flow cytometry for specific splenocyte cell populations. The percentage of T cells was determined by analyzing splenocytes bearing the cell surface markers CD3 (T cells), CD4 (CD3^+^/CD4^+^; helper/inducer cells), and CD8 (CD3^+^/CD8^+^; suppressor/cytotoxic cells). Relative to young WT mice, the percentage of CD3^+^ T cells in splenocytes from aged WT mice was reduced (13%, *P* < .05), Table 1. Interestingly, this difference was more pronounced in splenocytes from aged IL-6 KO mice relative to cells from young IL-6 KO mice (24% reduction in CD3^+^ T cells, *P* < .001). An analysis of splenic CD3^+^/CD4^+^ helper/inducer T cells revealed no effects of aging or IL-6 on their percentage. However, CD3^+^/CD8^+^ suppressor/cytotoxic T cells were reduced in splenocytes from aged WT mice (25%, *P* < .05), relative to young WT mice. In addition, a marked (47%) decrease in CD3^+^/CD8^+^ suppressor/cytotoxic T cells was found in aged IL-6 KO animals, relative to splenocytes from young IL-6 KO mice. Analysis of B cell percentages in the spleen was also assessed by measuring B220^+^ splenocytes, Table 1. Spleens of young WT mice were composed of 40% B220^+^cells. This percentage was slightly increased by aging and IL-6 deficiency, however, these differences failed to reach statistical significance. Similarly, analysis of the F4/80^+^ macrophage population showed no effects of aging or IL-6 deficiency on their percentage.

### 3.3. Effects of Age and IL-6 on TLR4 Expression in Macrophages

TLR4 involvement in age-related defects in macrophage activation following LPS activation has been documented extensively (reviewed in [1, 2]). However, TLR4 levels in the context of aging and IL-6 deficiency are unknown. To analyze the expression of TLR4 in macrophages from aged IL-6 KO mice, splenic macrophages were examined by flow cytometry. As we reported previously [16, 17], F4/80^+^ macrophages from aged WT mice did not exhibit age-associated differences in surface TLR4 expression (23.0% in young WT and 24.7% in aged WT), Table 2. In F4/80^+^ macrophages from IL-6 KO mice, a similar percentage of TLR4^+^ cells was found relative to those from WT mice, regardless of age or IL-6 deficiency (24.6% in young IL-6 KO versus 23.6% in aged IL-6 KO). These results suggest that changes in TLR4 expression in macrophages are not responsible for the increase in proinflammatory cytokine production observed in macrophages from aged IL-6 KO mice.

### 3.4. Effects of Age and IL-6 on IL-6 Receptor Expression in Macrophages

*In vivo* as well as *in vitro* studies suggest that stimulation with IL-6 affects the expression of the IL-6 receptor [32, 33]. To analyze the effects of aging and IL-6 on the initial steps of IL-6 signaling, we measured surface levels of the IL-6 receptor in total spleen cell suspensions by flow cytometry. IL-6 signaling is initiated by binding of the cytokine to an 80-kDa glycoprotein *α* chain (IL-6Ra/CD126) [34]. F4/80^+^ macrophages from aged WT mice did not differ in the percentage of IL-6Ra expressing cells (Table 2) or the level of the surface expression as measured by mean fluorescent intensity (MFI) (data not shown) relative to those from young WT mice. In F4/80^+^ macrophages from IL-6 KO mice, similar levels of IL-6Ra were found relative to those from WT mice, regardless of age or IL-6 deficiency. Following binding of IL-6 to IL-6Ra, dimerization of signal-transducing glycoprotein 130 (gp130/CD130) leads to activation of the Janus kinase (JAK)/signal transducer and activator of transcription (STAT) 3 signal transduction pathway. As shown in Table 2, gp130 levels on F4/80^+^ macrophages from young or aged WT mice were similar. No effects of aging or IL-6 were found in surface expression of gp130 in cells from IL-6 KO mice.

## 4. Discussion

In this report, we analyzed the effects of aging and IL-6 on cytokine production by macrophages *in vitro*. Our results add to published data [15–19] showing decreased synthesis and release of the proinflammatory cytokines, TNF*α*, IL-*1* *β*, IL-6, and IL-12, in macrophages from aged WT mice compared to those from young WT animals after LPS stimulation. In addition, we have found for the first time that knocking out IL-6 restores proinflammatory cytokine production by macrophages from aged mice to the levels of macrophages from young WT mice. When compared to aged WT mice, splenocytes from aged IL-6 KO mice had similar age-related decreases in the relative abundance of CD3^+^ T cells, CD3^+^/CD4^+^ T cells, and Ly49c^+^ NK cells, however, a more pronounced decrease was found in CD3^+^/CD8^+^ T cells. No effects of aging or IL-6 deficiency were found on the percentage of F4/80^+^ macrophages and CD19^+^ B cell populations, neither in the surface expression of TLR4, nor the components of the IL-6 receptor, IL-6Ra, and gp130. Overall, our results indicate that IL-6 plays a role in regulating the age-related defects in macrophages through alteration of proinflammatory cytokines. Additional investigation is needed to clarify the cellular mechanisms involved in such effects.

Macrophages show the impact of advanced aged in many of their biological properties including cytokine production (recently reviewed in [1, 2]). In general, when cultured *in vitro *with LPS, macrophages from aged mice produce lower levels of proinflammatory cytokines than comparably stimulated cells from young mice [15–19]. As previously reported, compared to macrophages from young WT mice, macrophages from aged WT mice had decreased production of TNF*α* and IL-6. IL-1*β* and IL-12 production after LPS stimulation also was reduced in macrophages from aged WT mice. These results are coincident with findings from Chelvarajan and collaborators and are independent of the source used to isolate the macrophages (plastic adherence utilized in current study versus positive selection from unfractionated splenocytes using magnetic sorting with CD11b microbeads used by Chelvarajan and collaborators [18]). Overall, our findings agree with published literature in demonstrating decreased cytokine production in macrophages from aged WT mice. 

Interleukin-6 deficiency in aged mice restored the cytokine production profile of macrophages to that of young WT mice. Age-related alterations in the function of macrophages result from the combination of intrinsic and extrinsic defects and possibly the consequence of the complex interactions with other cell types or the aged milieu [22]. Our results, added to the previous findings [24, 25, 35–37], suggest that altered production of cytokines in macrophages from aged mice results in part from changes in the expression of extrinsic factors. Cells from young IL-6 KO mice had significantly lower levels of IL-1*β*, TNF*α*, and IL-12 when incubated with LPS relative to their WT counterparts. We still don't have an explanation for these results, however, it can be speculated that a modification in the steady-state of components of cellular pathways shared between the IL-6 signaling cascade and proinflammatory cytokine expression (i.e., Erk and AP-1) may be a suitable common link. Additional studies will clarify this issue.

Interleukin-6 supports the growth of splenic T and B lymphocytes [30, 31]. It was therefore our interest to study if the effects of aging and the lack of IL-6 in cytokine production by macrophages could be attributed to changes in the numbers of these cells in the spleen. While some of the splenocyte populations we tested showed age-related decreases in cytokine release, regardless of the presence of IL-6, we failed to find changes for F4/80^+^ macrophages between young WT mice and young IL-6 KO mice. Unexpectedly, a more pronounced decrease was found in CD3^+^/CD8^+^ T cells (25% in aged WT versus 47% in aged IL-6 KO, *P* < .05). Our results agree with those from Kopf and collaborators indicating no effects of IL-6 deficiency on the B cell compartment in the bone marrow and spleen of young IL-6 KO mice, with normal expression of B220, IgM, IgD, and CD23 [38]. In this same study the expression of the T cell receptor *α*, *β*, *γ*, *δ* chains, CD4, CD8, CD44 (Pgpl), and CD24 (HSA) in splenocytes from young IL-6 KO mice was unchanged [38]. Kopf et al. also reported a 30% to 50% reduction in the total number of splenocytes in young IL-6 KO mice compared to young controls [38]. Similarly, we observed a 40% reduction in the number of splenocytes in young IL-6 KO mice as well as in their concanavalin-A induced proliferative response relative to young WT mice (data not shown). Overall, these results stress the involvement of IL-6 in the expansion and function of specific lymphoid cell subsets. Further investigation will help to clarify the effects of IL-6 on some of the age-related T cell defects, such as lymphocyte activation.

The impact of aging on cell signaling in macrophages has been analyzed by several groups (reviewed in [39]). Renshaw and collaborators linked defective production of cytokines in macrophages from aged mice following LPS stimulation to a decline in all subclasses of mRNA for TLRs as well as decreased surface expression of TLR4 on cells from the aged mice when compared with those from young mice [15]. However, two independent studies from separate groups failed to show age-dependent differences in cell-surface expression of TLR4 [16, 18]. In our study, we showed similar surface expression of TLR4 in macrophages from aged WT mice [16, 18]. TLR4 expression was not affected by either aging or lack of IL-6 in macrophages from senescent IL-6 KO mice. Previously, we reported that aged IL-6 KO mice had similar serum levels of lipopolysaccharide-binding protein (LPB) relative to aged WT mice [10]. Neither advanced age nor IL-6 deficiency modified the ability to induce production of this protein *in vivo* after an inflammatory challenge [10]. Therefore, age or IL-6 deficiency does not appear to affect surface expression of the initial molecules involved in LPS signal transduction. This conclusion also seems to be valid for the components of the IL-6 receptor whose levels remained unchanged in macrophages regardless of age or lack of IL-6. In humans, there was a significant increase of soluble IL-6 receptor until around age 70 and a gradual decline in its levels after age 70 [40]. In mice, administration of IL-6 triggered an upregulation of the components of the IL-6 receptor in several tissues in mice embryos early during their development [32]. However, aged MRL/lpr mice, genetically predisposed to the development of autoimmune diseases and reported to have elevated levels of IL-6 and sIL-6R levels, had a marked downregulation of gp130 in splenic T cells [33]. Our findings differ from the published literature and suggest that up or downregulation in expression of the IL-6 receptor seen in other models is unlikely to operate in macrophages when IL-6 is “chronically absent” and maybe alternative compensatory mechanisms are operating in cells from aged IL-6 KO mice. Most likely in our aged IL-6 KO mice, the mechanisms responsible for age-dependent effects of IL-6 in LPS-mediated cell activation observed in macrophages may originate at the intracellular level, such as that which has been observed in macrophages from aged WT mice. Examples of this may include defects in intracellular activation cascades [15–17, 41].

There is only a handful of publications on the effects of IL-6 and aging on specific cell types involved in inflammatory responses. Most of the work has been performed in mouse models of systemic inflammatory responses following burn injury, during sepsis, or after LPS administration. In one of these studies, we analyzed the effects of aging and IL-6 on the hepatic inflammatory response in two models of systemic injury: dorsal scald (burn) injury versus intraperitoneal LPS administration. Evidence obtained from histological observation showed comparable numbers of polymorphonuclear cells (PMNs) in the livers of burn-injured mice regardless of age or IL-6 deficiency. However, increased hepatic neutrophils were seen in aged wild type (WT) mice given LPS relative to young WT mice given LPS. Accumulation of hepatic PMNs was drastically reduced in aged IL-6 KO mice given LPS as compared with LPS-exposed aged WT mice. These results suggest a role of PMNs in the—insult specific—hepatic injury mediated by IL-6 in aged animals [11]. Starr and collaborators examined the expression of IL-6 in various tissues in aged WT mice during LPS-induced systemic inflammation. Among the different tissues tested, white adipose tissue from epididymal fat pad expressed the highest level of IL-6 mRNA in both young and aged mice with a 5.5-fold higher level in the aged. Immunohistochemistry revealed that LPS-induced IL-6 expression was associated to both the adipocytes and stromal cells. Aged IL-6 KO mice exhibited reduced mortality to LPS suggesting a deleterious effect of IL-6 overexpression in the aged [12]. These results suggest that increased vulnerability to systemic inflammation with age is due in part to augmented IL-6 production by the adipose tissue. Overall, a role for other cells, besides macrophages, in work documenting advanced age and IL-6 *in vivo* is suggested by the two publications showed above [11, 12]. This report is the first to analyze the effects of aging and IL-6 on cytokine production in macrophages in vitro. Additional research will clarify the cellular mechanisms involved in our findings as well as the effects of aging and IL-6 on other cell types.

## Figures and Tables

**Table 1:** Effects of age and IL-6 on splenocyte cell populations.

	Cells (%)			
	WT		IL-6 KO	
	Young	Aged	Young	Aged
CD3^+^ T Cells	47.1 ± 1.1	41.1 ± 1.1*	48.3 ± 1.1	36.9 ± 0.9*
CD3^+^/CD4^+^ T Cells	34.1 ± 0.9	31.0 ± 0.8	32.9 ± 0.8	30.1 ± 1.7
CD3^+^/CD8^+^T Cells	13.6 ± 0.9	10.2 ± 0.7*	16.6 ± 0.4**	8.8 ± 0.2*
Ly49c^+^NK Cells	5.4 ± 0.1	4.2 ± 0.3*	5.4 ± 0.2	4.1 ± 0.2*
F4/80^+^Macrophages	15.9 ± 2.1	12.6 ± 1.2	14.1 ± 2.3	13.3 ± 0.9
CD19^+^B cells	41.1 ± 1.5	43.4 ± 1.2	43.6 ± 2.3	46.9 ± 0.9

Splenocytes were incubated with anti-CD3, CD4, CD8, Ly49c, F4/80, or CD19 antibodies and analyzed by flow cytometry. Data are shown as mean ± SEM of 4-5 mice per group. *P < .05 from aged matched control. **P < .05 from young WT.

**Table 2:** Percentage of *T* *L* *R*4^+^, *I* *L*-6*R* *a* ^+^, and *G* *p*130^+^ in *F*4/80^+^ splenocytes.

	F4/80^+^ cells (%)			
	WT		IL-6 KO	
	Young	Aged	Young	Aged
TLR4^+^	23.0 ± 2.8	24.7 ± 1.7	24.6 ± 2.5	23.6 ± 0.4
IL-6Ra^+^	61.4 ± 1.3	60.1 ± 1.5	59.1 ± 1.1	60.2 ± 2.2
gp130^+^	35.6 ± 6.3	27.2 ± 2.0	29.9 ± 4.1	31.2 ± 2.4

Splenocytes were incubated with anti-F4/80 and anti-TLR4 antibodies or with anti-F4/80, anti IL-6Ra, and anti-Gp130 antibodies and analyzed by flow cytometry. Data are shown as mean ± SEM of 4-5 mice per group.

## Abbreviations

IL: Interleukin
IL-6Ra: IL-6 receptor antagonist
KO: Knockout
LPS: Lipopolysaccharide
MFI: Mean fluorescence intensity
TLR4: Toll-like receptor 4
TNF*α*: Tumor necrosis factor alpha
WT: Wild type.

## References

[1] Gomez CR, Nomellini V, Faunce DE, Kovacs EJ (2008). Innate immunity and aging. *Experimental Gerontology*.

[2] Kovacs EJ, Palmer JL, Fortin CF, Fülöp T, Goldstein DR, Linton P-J (2009). Aging and innate immunity in the mouse: impact of intrinsic and extrinsic factors. *Trends in Immunology*.

[3] Linton PJ, Dorshkind K (2004). Age-related changes in lymphocyte development and function. *Nature Immunology*.

[4] Bruunsgaard H (1999). A high plasma concentration of tnf-*α* is associated with dementia in centenarians. *Journals of Gerontology. Series A*.

[5] Yoshikawa TT (2000). Epidemiology and unique aspects of aging and infectious diseases. *Clinical Infectious Diseases*.

[6] Ginaldi L, Loreto MF, Corsi MP, Modesti M, De Martinis M (2001). Immunosenescence and infectious diseases. *Microbes and Infection*.

[7] Trzonkowski P, Myśliwska J, Godlewska B (2004). Immune consequences of the spontaneous pro-inflammatory status in depressed elderly patients. *Brain, Behavior, and Immunity*.

[8] Franceschi C, Bonafè M, Valensin S (2000). Inflamm-aging. An evolutionary perspective on immunosenescence. *Annals of the New York Academy of Sciences*.

[9] Ershler WB, Keller ET (2000). Age-associated increased interleukin-6 gene expression, late-life diseases, and frailty. *Annual Review of Medicine*.

[10] Gomez CR, Goral J, Ramirez L, Kopf M, Kovacs EJ (2006). Aberrant acute-phase response in aged interleukin-6 knockout mice. *Shock*.

[11] Gomez CR, Nomellini V, Baila H, Oshima K, Kovacs EJ (2009). Comparison of the effects of aging and IL-6 on the hepatic inflammatory response in two models of systemic injury: Scald injury versus I.P. LPS administration. *Shock*.

[12] Starr ME, Evers BM, Saito H (2009). Age-associated increase in cytokine production during systemic inflammation: adipose tissue as a major source of IL-6. *Journals of Gerontology. Series A*.

[13] Underhill DM, Ozinsky A (2002). Phagocytosis of microbes: complexity in action. *Annual Review of Immunology*.

[14] Albright JF, Albright JW (2003). Senescence of natural/innate resistance to infection. *Aging, Immunity, and Infection*.

[15] Renshaw M, Rockwell J, Engleman C, Gewirtz A, Katz J, Sambhara S (2002). Cutting edge: impaired toll-like receptor expression and function in aging. *Journal of Immunology*.

[16] Boehmer ED, Goral J, Faunce DE, Kovacs EJ (2004). Age-dependent decrease in Toll-like receptor 4-mediated proinflammatory cytokine production and mitogen-activated protein kinase expression. *Journal of Leukocyte Biology*.

[17] Boehmer ED, Meehan MJ, Cutro BT, Kovacs EJ (2005). Aging negatively skews macrophage TLR2- and TLR4-mediated pro-inflammatory responses without affecting the IL-2-stimulated pathway. *Mechanisms of Ageing and Development*.

[18] Chelvarajan RL, Collins SM, Van Willigen JM, Bondada S (2005). The unresponsiveness of aged mice to polysaccharide antigens is a result of a defect in macrophage function. *Journal of Leukocyte Biology*.

[19] Chelvarajan RL, Liu Y, Popa D (2006). Molecular basis of age-associated cytokine dysregulation in LPS-stimulated macrophages. *Journal of Leukocyte Biology*.

[20] Krabbe KS, Bruunsgaard H, Hansen CM (2001). Ageing is associated with a prolonged fever response in human endotoxemia. *Clinical and Diagnostic Laboratory Immunology*.

[21] Krabbe KS, Pedersen M, Bruunsgaard H (2004). Inflammatory mediators in the elderly. *Experimental Gerontology*.

[22] Gomez CR, Boehmer ED, Kovacs EJ (2005). The aging innate immune system. *Current Opinion in Immunology*.

[23] Stout RD, Jiang C, Matta B, Tietzel I, Watkins SK, Suttles J (2005). Macrophages sequentially change their functional phenotype in response to changes in microenvironmental influences. *Journal of Immunology*.

[24] Wu D, Ren Z, Pae M (2007). Aging up-regulates expression of inflammatory mediators in mouse adipose tissue. *Journal of Immunology*.

[25] Gómez CR, Acuña-Castillo C, Nishimura S (2006). Serum from aged F344 rats conditions the activation of young macrophages. *Mechanisms of Ageing and Development*.

[26] Kopf M, Baumann H, Freer G (1994). Impaired immune and acute-phase responses in interleukin-6-deficient mice. *Nature*.

[27] Faunce DE, Gregory MS, Kovacs EJ (1998). Glucocorticoids protect against suppression of T cell responses in a murine model of acute ethanol exposure and thermal injury by regulating IL-6. *Journal of Leukocyte Biology*.

[28] Ershler WB, Sun WH, Binkley N (1993). Interleukin-6 and aging: Blood levels and mononuclear cell production increase with advancing age and in vitro production is modifiable by dietary restriction. *Lymphokine and Cytokine Research*.

[29] Daynes RA, Araneo BA, Ershler WB, Maloney C, Li G-Z, Ryu S-Y (1993). Altered regulation of IL-6 production with normal aging: possible linkage to the age-associated decline in dehydroepiandrosterone and its sulfated derivative. *Journal of Immunology*.

[30] Kishimoto T (2005). Interleukin-6: from basic science to medicine—40 years in immunology. *Annual Review of Immunology*.

[31] Puri RK, Leland P (1992). Systemic administration of recombinant interleukin-6 in mice induces proliferation of lymphoid cells in vivo. *Lymphokine and Cytokine Research*.

[32] Saito M, Yoshida K, Hibi M, Taga T, Kishimoto T (1992). Molecular cloning of a murine IL-6 receptor-associated signal transducer, gp130, and its regulated expression in vivo. *Journal of Immunology*.

[33] Wang X-J, Taga T, Yoshida K, Saito M, Kishimoto T, Kikutani H (1998). gp130, the cytokine common signal-transducer of interleukin-6 cytokine family, is downregulated in T cells in vivo by interleukin-6. *Blood*.

[34] Lutticken C, Wegenka UM, Yuan J (1994). Association of transcription factor APRF and protein kinase Jak1 with the interleukin-6 signal transducer gp130. *Science*.

[35] Arthur WT, Vernon RB, Sage EH, Reed MJ (1998). Growth factors reverse the impaired sprouting of microvessels from aged mice. *Microvascular Research*.

[36] De Cabo R, Cabello R, Rios M (2004). Calorie restriction attenuates age-related alterations in the plasma membrane antioxidant system in rat liver. *Experimental Gerontology*.

[37] De Cabo R, Fürer-Galbán S, Anson RM, Gilman C, Gorospe M, Lane MA (2003). An in vitro model of caloric restriction. *Experimental Gerontology*.

[38] Kopf M, Ramsay A, Brombacher F (1995). Pleiotropic defects of IL-6-deficient mice including early hematopoiesis, T and B cell function, and acute phase responses. *Annals of the New York Academy of Sciences*.

[39] Gomez CR, Nomellini V, Boehmer ED, Kovacs EJ (2007). Signal transduction of the aging innate immune system. *Current Immunology Reviews*.

[40] Giuliani N, Sansoni P, Girasole G (2001). Serum interleukin-6, soluble interleukin-6 receptor and soluble gp130 exhibit different patterns of age- and menopause-related changes. *Experimental Gerontology*.

[41] Yoon P, Keylock KT, Hartman ME, Freund GG, Woods JA (2004). Macrophage hypo-responsiveness to interferon-*γ* in aged mice is associated with impaired signaling through Jak-STAT. *Mechanisms of Ageing and Development*.

